# Isolation of Lagos Bat Virus from Water Mongoose

**DOI:** 10.3201/eid1212.060514

**Published:** 2006-12

**Authors:** Wanda Markotter, Ivan Kuzmin, Charles E. Rupprecht, Jenny Randles, Claude T. Sabeta, Alexander I. Wandeler, Louis H. Nel

**Affiliations:** * University of Pretoria, Pretoria, South Africa;; † Centers for Disease Control and Prevention, Atlanta, Georgia, USA;; ‡ Allerton Veterinary Laboratory, Pietermaritzburg, South Africa;; § Onderstepoort Veterinary Research Institute, Pretoria, South Africa;; ¶ Canadian Food Inspection Agency, Nepean, Ontario, Canada

**Keywords:** Lagos bat virus, rabies, rabies-related viruses, lyssaviruses, nucleoprotein, Herpestidae, mongoose, Atilax paludinosus, South Africa, cytochrome b, pathogenesis, phylogeny, research

## Abstract

A genotype 2 lyssavirus, Lagos bat virus (LBV), was isolated from a terrestrial wildlife species (water mongoose) in August 2004 in the Durban area of the KwaZulu-Natal Province of South Africa. The virus isolate was confirmed as LBV by antigenic and genetic characterization, and the mongoose was identified as *Atilax paludinosus* by mitochondrial cytochrome *b* sequence analysis. Phylogenetic analysis demonstrated sequence homology with previous LBV isolates from South African bats. Studies performed in mice indicated that the peripheral pathogenicity of LBV had been underestimated in previous studies. Surveillance strategies for LBV in Africa must be improved to better understand the epidemiology of this virus and to make informed decisions on future vaccine strategies because evidence is insufficent that current rabies vaccines provide protection against LBV.

## Abstract 2

One-sentence summary for table of contents: Lagos bat virus from water mongoose showed strong sequence homology with other Lagos bat virus isolates from South Africa.

Lagos bat virus (LBV) belongs to the genus Lyssavirus in the family Rhabdoviridae. The prototype lyssavirus genotype and species, rabies virus (RABV), has a single, continuous, negative-strand RNA of ≈12,000 nt that codes for 5 proteins: nucleoprotein, matrix protein, phosphoprotein, glycoprotein, and polymerase (*1*). The Lyssavirus genus was created after isolation of several viruses in Africa and Europe that were related to, but serologically distinct from, RABV (*2*).

Seven genotypes (gts) or species in this genus are recognized (*3*), and diversity may expand with the addition of new isolates from Eurasia (*4*), which are tentative species in the Lyssavirus genus. RABV (gt1) is distributed worldwide, Australian bat lyssavirus (gt7) has only been identified in Australia, and European bat lyssavirus 1 (EBLV-1) (gt5) and European bat lyssavirus 2 (EBLV-2) (gt6) have been found only in Europe. Lagos bat virus (LBV) (gt2), Mokola virus (gt3), and Duvenhage virus (gt4) have been found only in Africa.

Recognized lyssavirus genotypes are divided into 2 serologically, pathogenically, and genetically distinct phylogroups (*5*). One phylogroup consists of Mokola virus and LBV (group II), while all other genotypes are in group I. Members of phylogroup I are reported to be pathogenic for mice when introduced intramuscularly and intracerebrally. In contrast, members of phylogroup II are believed to be pathogenic in mice only when introduced by the intracerebral (i.c.) route (*5*). Commercial vaccine strains belong to gt1 (RABV) phylogroup 1, and these vaccines provide protection against RABV and all the other members of phylogroup I. However, laboratory data suggest that these vaccines (gt1 based) will not offer protection against lyssaviruses in the phylogroup II cluster (*6**,**7*). On the basis of criteria proposed for lyssavirus phylogroups, West Caucasian bat virus could be considered an independent phylogroup III because of genetic distance and absence of serologic cross-reactivity with phylogroup I and II viruses (*7*).

LBV was first isolated from a fruit bat (Eidolon helvum) in 1956 on Lagos Island in Nigeria (*2**,**8*). Fourteen isolations of this virus have been reported throughout Africa, including 8 in South Africa (*9*). Most LBV isolates were obtained from bats; 2 were from domestic cats (*10**,**11*), and 1 was from a domestic dog in Ethiopia (*12*). LBV has never been isolated from any terrestrial wildlife species.

Globally and throughout Africa, RABV (gt1) is the most common lyssavirus. In southern Africa, 2 biotypes of RABV are recognized (*13**,**14*): the canid biotype, which mainly circulates among dogs, jackals, and bat-eared foxes, and the mongoose biotype, which is well adapted and unique to mongooses in southern Africa (*15*). RABV is responsible for all mongoose rabies cases in Africa. In South Africa, the principal vector of the mongoose biotype is the yellow mongoose (Cynictis penicillata), but RABV has been reported in other mongoose species, such as slender (Galerella sanguinea), water (Atilax paludinosus), small gray (Galerella pulverulenta), banded (Mungos mungo), selous (Paracynictis selousi), dwarf (Helogale parvula), and white-tailed (Ichneumia albicauda) mongooses. Mongoose rabies in South Africa commonly occurs in the central highveld regions (*15**,**16*), whereas KwaZulu-Natal Province, which is located on the east coast of South Africa, is associated with epizootic canid rabies in domestic dogs; mongoose rabies is not reported in this province.

We report the first identification of LBV in a wildlife terrestrial species, A. paludinosus, commonly known as the water or marsh mongoose. The mongoose species was identified by generation and analysis of cytochrome b sequencing data. We characterized this LBV isolate by antigenic typing with antinucleocapsid monoclonal antibodies, sequencing of the nucleoprotein gene, and peripheral pathogenicity in laboratory mice in comparison with other LBV isolates from South Africa and a bat RABV isolate from North America.

## Materials and Methods

### Sample Collection

In August 2004, a brain sample from a suspected rabid mongoose was submitted to the Allerton Veterinary Institute in Pietermaritzburg, KwaZulu-Natal, South Africa. The mongoose was captured by the Society for the Prevention of Cruelty to Animals in a marshy valley in a residential area in Westville near Durban after the mongoose displayed abnormal behavior. The animal was disorientated, attacked inanimate objects, and alternated between being friendly and aggressive. Only the brain of the animal was submitted for testing; the carcass was not preserved. The mongoose species was not identified.

### Virus Characterization

Lyssavirus antigen was detected by the standard fluorescent antibody test (FAT) (*17*), with modifications, by using a polyclonal fluorescein isothiocyanate–conjugated immunoglobulin (Rabies Unit, Onderstepoort Veterinary Institute, Pretoria, South Africa) that could detect all lyssavirus genotypes. Virus isolation was performed by using the i.c. mouse inoculation test in suckling mice (*18*). Antigenic typing was performed by using the indirect fluorescent antibody test with a panel of 16 antinucleocapsid monoclonal antibodies (N-MAbs) (Centre of Expertise for Rabies, Canadian Food Inspection Agency, Nepean, Ontario, Canada) as previously described (*19*). Genetic characterization was based on sequencing of the entire nucleoprotein (N) gene.

Briefly, total RNA was extracted from infected brain material with Trizol (Invitrogen, Croningen, the Netherlands) according to the manufacturer's instructions. Complementary DNA was produced by a reverse transcription reaction by using an oligonucleotide primer specific for the noncoding messenger RNA of the lyssavirus genome (Lys001: 5´-ACGCTTAACGAMAAA-3´ position 1–15 according to the Pasteur virus [PV] RABV genome, GenBank accession no. M13215). Complementary DNA was amplified with a PCR by using different combinations of the oligonucleotide primers Lys001, LagNF (*9*), 550B (5´-GTRCTCCARTTAGCRCACAT-3´, position 647–666 according to the PV genome), and 304 (5´-TTGACAAAGATCTTGCTCAT-3´, position 1514–1533 according to the PV genome) as described elsewhere (*20*). The PCR products were visualized after electrophoresis on a 2% agarose gel and purified by using the Wizard PCR Preps DNA Purification System (Promega, Madison, WI, USA). The purified products were sequenced with the BigDye Termination Cycle Sequencing Ready Reaction Kit 1.1 (Applied Biosystems, Foster City, CA, USA), according to the manufacturer's protocol, with subsequent analysis on an Applied Biosystems 377 DNA automated sequencer.

### Sequence Analysis

DNA sequencing information was compared with nucleoprotein sequence information for other lyssavirus genotypes in GenBank, as well as with nucleoprotein sequencing data obtained during this study from previous LBV isolates in South Africa from Epomophorus whalbergi fruit bats in 1980 (*21*), 1982 (*11*), 2003 (*9*), and 2004 (*9*), by using the same method as described above. CLUSTAL W (*22*) was used to produce sequence alignments and generate a neighbor-joining phylogenetic tree. A graphic representation of the tree was constructed with the TREEVIEW program (*23*).

### Virus Pathogenicity

Two LBV isolates from South Africa (LBVSA2004) (9) and the LBV mongoose isolate described in this report (Mongoose2004), as well as a North American bat RABV (Myotis spp. variant, isolated in Washington, USA, 2004), were injected into 4-week-old inbred ICR mice (5 mice/group) by different routes. The i.c. 50% lethal dose (LD_50_) was determined by titration of the virus suspension injected into 4-week-old ICR mice by the i.c. route. Thereafter, 4-week-old ICR mice were injected with 30 μL of 10^3^ LD_50_ of each virus by the i.c. route and 30 μL of 10^5^ LD_50_ of each virus by the intramuscular (i.m.) route. Virus inoculum was prepared by 1 i.c. passage of the original mongoose brain material in suckling mice.

### Species Identification of the LBV-infected Mongoose

Because the mongoose carcass was destroyed, we attempted to accurately identify the animal by using DNA sequencing analyses of the mitochondrial cytochrome b region of mongoose genomic DNA obtained from the brain sample. The mitochondrial cytochrome b region has been used to characterize relationships between mongoose species (*24*). Genomic DNA was extracted from mongoose brain by using the DNeasy Blood and Tissue kit (Qiagen, Hilden, Germany), followed by PCR conducted according to the method of Veron et al. (*24*). PCR products were purified by using the Wizard SV PCR and gel purification kit (Promega) and sequenced by using the BigDye Termination Cycle Sequencing Ready Reaction Kit 3.1 (Applied Biosystems) according to the manufacturer's protocol, with subsequent analysis on an Applied Biosystems 3100 DNA automated sequencer. A DNA sequence of 893 bp of the cytochrome b gene was compared with cytochrome b sequences for mongooses available in GenBank by the same method as described earlier for the analysis of LBV nucleoprotein gene sequences.

## Results

### Virus Characterization and DNA Sequence Analysis

FAT performed on mongoose brain material showed a positive reaction for lyssavirus antigen. During the mouse inoculation test, suckling mice died 9 days after i.c. injections with mongoose brain suspensions. FAT of the suckling mouse brains showed a positive reaction for lyssavirus antigen. The isolate reacted with N-MAb 38HF2, which is an antibody that reacts with all lyssaviruses tested, and with the antibody N-MAb M612, which is highly specific for LBV and does not react with any other lyssaviruses tested. These findings indicate that the new isolate belongs to LBV (Table).

**Table:** Immunofluorescence patterns of 16 monoclonal antibodies with nucleoprotein of a new Lagos bat virus isolate from a mongoose in South Africa (Mongoose2004) compared with antigenic patterns of other South African lyssaviruses*

Antibody	RABV (canid biotype)	RABV (mongoose biotype)	LBV (bat isolates)	MOKV	DUVV	LBV (Mongoose 2004)
1C5	–	–	–	–	–	–
26AB7	+	Variable	–	–	–	–
26BE2	+	Variable	–	–	–	–
32GD12	Variable	Variable	–	–	–	–
38HF2	+	+	+	+	+	+
M612	–	–	+	–	–	+
M837	–	–	–	–	+	–
M850	–	Variable	–	–	+	–
M853	+	–	–	–	+	–
M1001	–	–	–	+	–	–
M1335	–	Variable	–	Variable	–	–
M1386	–	+	–	–	–	–
M1400	–	Variable	–	–	–	–
M1407	+	Variable	–	–	–	–
M1412	+	Variable	–	–	–	–
M1494	–	Variable	–	–	+	–

*Typical immunofluorescence antibody patterns observed for all lyssavirus genotypes present in Africa are included. RABV, rabies virus; LBV, Lagos bat virus; MOKV, Mokola virus; DUVV, Duvenhage virus; –, no fluorescence; +, fluorescence.

**Figure 1:**
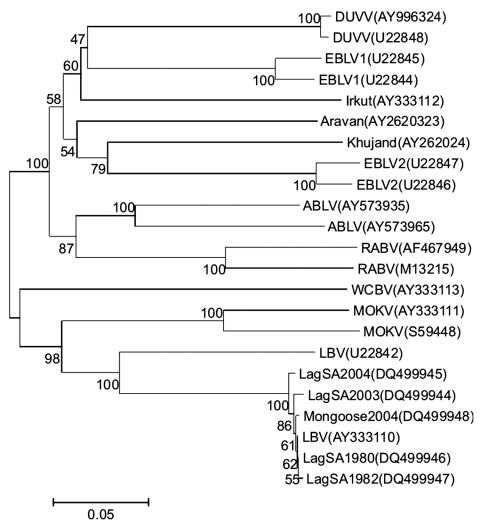
Neighbor-joining phylogenetic tree comparing nucleotide sequences of the entire nucleoprotein gene (1,350 nt) of a new Lagos bat virus (LBV) isolate from a mongoose in South Africa (Mongoose2004) and representative sequences of all other genotypes of lyssaviruses. Branch lengths are drawn to scale, and bootstrap values for 1,000 replicates are shown for the nodes. Accession numbers for all sequences available from GenBank and full-length nucleoprotein sequences of other LBV isolates from South Africa (1980, 1982, 2003, and 2004) are also included. DUVV, Duvenhage virus; EBLV, European bat lyssavirus; ABLV, Australian bat lyssavirus; RABV, rabies virus; WCBV, West Caucasian bat lyssavirus; MOKV, Mokola virus.

A reverse transcription–PCR method followed by a cycle sequencing method was used to amplify and determine the nucleic acid sequence of the entire nucleoprotein-encoding gene of the new putative LBV isolate. Phylogenetic analysis indicated that the new isolate clusters together with LBV isolates from South Africa and with LBV isolates from Nigeria (*2*) and Ethiopia (*12*) (Figure 1). LBV isolates from South Africa, including the new mongoose isolate of LBV, showed high nucleotide sequence identity with each other (99.1%–99.7%), compared with low sequence identity (≈82%) with the LBV isolate from Nigeria. The LBV isolate from Ethiopia (isolated from a dog; GenBank accession no. AY333110) showed 99.1%–99.9% nucleotide sequence homology with the South African LBV isolates. This result warrants further investigation of the DNA sequence identity of the Ethopian LBV isolate.

### Virus Pathogenicity

**Figure 2:**
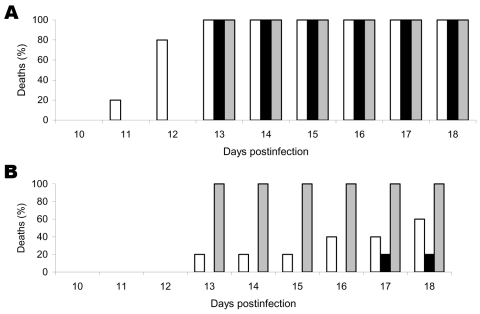
Pathogenicity of genotype 2 (LBVSA2004 [white bars] and Mongoose2004 [black bars]) and genotype 1 (gray bars) lyssaviruses in mice. Results are percentages of dead animals observed for a specific period. Mice were observed for 56 days, but no deaths occurred after 18 days. A) Deaths after intracerebral injection of 10^3^ 50% lethal doses (LD_50_). B) Deaths after intramuscular injections of 10^5^ LD_50_.

Genotypes 1 and 2 viruses were pathogenic for mice by the i.c. and i.m. routes of injection (Figure 2). A similar death rate was observed for both genotypes (100%) after i.c. injection of equal amounts of virus (10^3^ LD_50_ dose). Although the LBV isolates were lethal to mice when 10^5^ LD_50_ was injected intramuscularly, they were less efficient than the RABV isolate. Of mice injected with the LBV isolate from the mongoose, 20% died; 60% of the mice died after injection with the LBVSA2004 isolate from the fruit bat E. whalbergi. However, the RABV isolate showed 100% lethality in mice.

### Species Identification of the LBV-infected Mongoose

**Figure 3:**
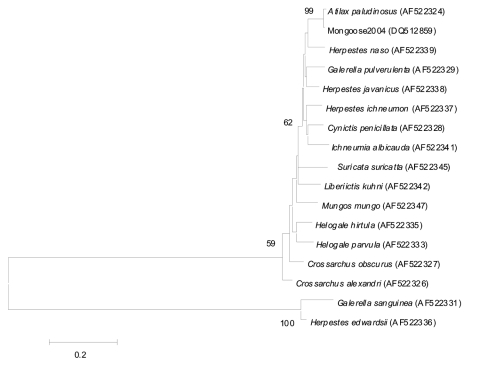
Neighbor-joining phylogenetic tree comparing 893 bp of the cytochrome *b* region of *Herpestidae* family sequences available in GenBank. Sequence obtained from the Lagos bat virus–infected mongoose (Mongoose2004) is 98% identical to the known cytochrome *b* sequences of *Atilax paludinosus* (water mongoose). GenBank accession numbers are indicated on the phylogenetic tree, and bootstrap values were determined with 1,000 replicates.

Analysis of 893 bp of the cytochrome b gene obtained from mongoose brain indicated that the infected animal shared a 98% DNA nucleotide sequence homology with the African water mongoose (A. paludinosus) (Figure 3). Water mongooses are solitary and mainly nocturnal mammals, but they may also be active during the day. These animals live near water in areas with sufficient bush cover and have been found throughout sub-Saharan Africa (*25*).

## Discussion

Isolation of LBV from terrestrial wildlife serves as further confirmation of our lack of understanding of the incidence and host range of lyssaviruses in Africa. Poor surveillance of rabies-related viruses and poor diagnostic capability in most of Africa are large contributors to our lack of information and the obscurity of the African lyssaviruses. The fluorescent antibody test used as a diagnostic test for rabies can only indicate the presence of lyssavirus antigens and cannot distinguish between lyssavirus genotypes. To identify a lyssavirus precisely, antigenic typing or genetic characterization is necessary, but these techniques are beyond the capability of most laboratories responsible for rabies diagnostics in Africa. Our phylogenetic analysis indicated a strong nucleoprotein sequence homology between LBV isolates from South Africa. Geographic partitioning is a well-known characteristic of RABV epidemiology worldwide. The strong sequence homology we observed may result from the defined geographic location from which all LBV isolates were obtained.

Although cases in domestic animals have been recorded, no human cases of infection with LBV have been documented. However, cross-neutralization data obtained with human sera and in rodent models suggest that preexposure and postexposure treatments for rabies are not effective against LBV (*6**,**7*). The infected mongoose showed aggressive behavior and was captured in a populated residential area. Although the incidence of the rabies-related viruses seems to be low, human exposure to these viruses is possible. Results of pathogenicity experiments indicated that death can occur from the i.c. and i.m. routes of injection, although gt2 viruses showed lower lethality to mice when injected i.m.

Our results differ from those of another study (*5*), which reported that a gt2 virus was not pathogenic to mice when administered by the i.m. route at the same dose (3×10^5^ LD_50_) used in our experiment. What amount of virus is involved in natural infection is not known. Cumulatively, our results indicate that LBV may be a health risk for humans and other mammals, and future vaccine strategies against rabies in Africa should consider these possibilities. Although laboratory data suggest little cross-neutralization of LBV by rabies preexposure and postexposure vaccination (*7*), immune system components other than neutralizing antibodies may be involved in protection. Therefore, in the absence of an alternative vaccine, rabies vaccination and postexposure treatment should still be advised because of potential cross-reactivity.

This report demonstrated the value of cytochrome b DNA sequencing for accurately identifying the host in a rabies case. Diagnostic laboratories do not routinely receive the complete carcass of suspected rabid animals, and identification is dependent on reports of persons who captured the animal or removed its brain before submission to the diagnostic facility. Host identity is rarely a problem in domestic animals, but wildlife species show potential uncertainty, such as demonstrated in the case reported here. One important aspect of disease epidemiology is accurate information about the host species involved, which enables informed decisions to be made with regard to the epidemiologic patterns and potential threats to public and veterinary health.

Identification of the first case of LBV in a mongoose underscores the need for surveillance of rabies-related viruses and the need for accurate identification of lyssavirus genotypes even if the host involved is normally only associated with RABV. With respect to LBV, we have recently reported the likely persistence of this virus in pteropid bats in South Africa, which implicates continuous opportunity for spillover into terrestrial species (*9*). In determining the extent of risk to human and veterinary public health, it is important to establish the prevalence of LBV not only in bats but also in potential terrestrial animal vectors, to which mongoose species should be added, based on the finding in this report.

The origin of mongoose rabies in South Africa is not clear (*14*). Epidemiologic cycles among yellow mongooses and other Herpestidiae are well established and shown to be impossible to extinguish or control by the attempted eradication or control of vector and host density (*26*). With respect to more modern or scientific approaches, no vaccination strategy has been considered feasible in tackling this complicated and entrenched wildlife rabies epidemic. Mongoose rabies may have originated from a spillover event of a bat lyssavirus progenitor in an event similar to the spillover described in this report.
